# Real world treatment patterns among patients with painful diabetic peripheral neuropathy in the United States

**DOI:** 10.1093/pm/pnag055

**Published:** 2026-04-21

**Authors:** Nathaniel M Schuster, Sushanth Jeyakumar, Ann M Menzie, Moeen Panni, Nicole Princic, Meghan Moynihan, Charles E Argoff

**Affiliations:** UC San Diego Health, San Diego, CA, United States; Vertex Pharmaceuticals Incorporated, Boston, MA, United States; Vertex Pharmaceuticals Incorporated, Boston, MA, United States; Vertex Pharmaceuticals Incorporated, Boston, MA, United States; MarketScan by Merative, Ann Arbor, MI, United States; MarketScan by Merative, Ann Arbor, MI, United States; Albany Medical College, Albany, NY, United States

**Keywords:** diabetic neuropathy, neuropathic pain, drug therapy, medication adherence, healthcare administrative claims

## Abstract

**Objective:**

This study analyzes real-world treatment patterns among patients newly initiating treatment for painful diabetic peripheral neuropathy using a contemporary dataset in the United States.

**Methods:**

A retrospective study using the MarketScan database (January 1, 2016, to June 30, 2023) identified adults newly prescribed pain medication(s) used to treat neuropathic pain within 60 days of a diabetic peripheral neuropathy diagnosis. Patients with prior medication, conditions with similar treatment indications, cancer, or major surgery were excluded. Treatment patterns over a 12-month period and variable-length follow-up (four sequential treatment episodes) were captured, including treatment type, dosage, adherence, discontinuation (≥90-day gap), and switching.

**Results:**

Among 22 955 patients identified, 98.5% initiated monotherapy treatment for painful diabetic peripheral neuropathy. The majority initiated treatment with gabapentinoids (gabapentin 59.0%; pregabalin 5.3%), followed by opioids (tramadol 15.1%, oxycodone 7.0%), and antidepressants (duloxetine 5.2%). Among patients initiating gabapentin, pregabalin, or duloxetine, most were prescribed daily doses below recommended levels (79% gabapentin, 91% pregabalin, 61% duloxetine), and the majority (81%–96%) did not increase their dose. Adherence was low. Over 50% discontinued their initial treatment within 3 months, and approximately 75% discontinued in the 12-month follow-up period, with fewer than 25% switching to another medication. When evaluating treatment patterns across sequential treatment episodes, almost 65% discontinued all pain medications and did not receive subsequent treatment by the fourth treatment episode.

**Conclusions:**

High rates of treatment discontinuation and suboptimal adherence likely indicate challenges with tolerability and insufficient pain relief associated with current therapies for painful diabetic neuropathy. These findings emphasize the limitations of existing treatment options and highlight the unmet need for more effective and better-tolerated treatments for these patients.

## Teaser

This study analyzed U.S. claims data (2016–2023) to evaluate treatment patterns for painful diabetic peripheral neuropathy (pDPN) among 22,955 adults. Most patients (98.5%) initiated monotherapy, primarily with gabapentinoids, opioids, or antidepressants, often at subtherapeutic doses. Adherence was low, with over 50% discontinuing treatment within 3 months and ∼75% within 12 months. Fewer than 25% switched medications. These findings highlight the need for more effective, better-tolerated therapies to address the unmet needs in pDPN management.

## Introduction

As of 2021, approximately 40 million adults aged 18 years or older—nearly 15% of the United States (US) adult population—were living with diabetes, with an estimated 1.2 million new diagnoses annually.^1^ Diabetic peripheral neuropathy (DPN) is a highly prevalent complication that affects nearly 50% of people with diabetes, and of those, 30%–50% have pain associated with DPN.^2–5^ Painful DPN is characterized by burning, lancinating, tingling, and/or electric shock-like sensations, which may occur in combination and often worsen at night.^3^ Pain may also be amplified in response to painful stimuli (hyperalgesia), or triggered by normally non-painful stimuli (allodynia).^3^^,^^6^ Painful DPN has a significant impact on daily functioning and is associated with depression, sleep disturbance, decreased quality of life, and impaired work productivity.^3^^,^^7^ Given the increasing prevalence of painful DPN in the United States and its downstream consequences, effective therapeutic measures are essential.^3^^,^^8^

Managing pain associated with DPN is challenging. Currently there are three US Food and Drug Administration (FDA)-approved oral medications for neuropathic pain associated with DPN: pregabalin, duloxetine, and tapentadol extended release. One topical treatment, capsaicin 8% topical system, is approved specifically for foot pain associated with DPN, and several neuromodulation treatments are FDA-approved or FDA-cleared for painful DPN.^3^ Current published clinical guidelines also recommend the treatment of painful DPN with the use of additional off-label pharmacotherapies including gabapentin, serotonin-norepinephrine reuptake inhibitors (SNRIs), tricyclic antidepressants (TCAs), and/or sodium channel antagonists to reduce pain.^9^^,^^10^ Despite their use, opioids are not recommended by clinical guidelines for painful DPN due to limited evidence of long-term efficacy and the risks of severe long-term adverse consequences.^9^^,^^11^

Medications frequently used to treat neuropathic pain have suboptimal safety profiles and limited efficacy.^5^^,^^12^ Gabapentinoids, SNRIs, and TCAs have tolerability concerns, which may include side effects such as somnolence, dizziness, and peripheral edema, while opioids are associated with risk of opioid use disorder and many adverse drug events impacting multiple body systems.^13^ These factors may lead to lower than recommended dosing, discontinuation, and suboptimal adherence, impacting real-world treatment effectiveness.^14^ According to survey data, 58% of patients with painful DPN remain dissatisfied with existing pharmacotherapies. Among those dissatisfied, two-thirds required greater pain relief and almost half needed longer pain relief,^5^^,^^15^ suggesting that pain is inadequately managed and underscoring substantial unmet clinical needs.^14^

To improve treatment and outcomes for patients with painful DPN, understanding current real-world treatment patterns is essential. The objectives of this study were to examine treatment patterns among adults newly initiating pain medications to manage painful DPN using contemporary real-world data. The study builds upon prior research published in 2015, by analyzing treatment patterns both within the first 12 months following treatment initiation and across sequential treatment episodes over a variable-length follow-up period.^14^

## Methods

### Study design and data source

**Figure 1:**
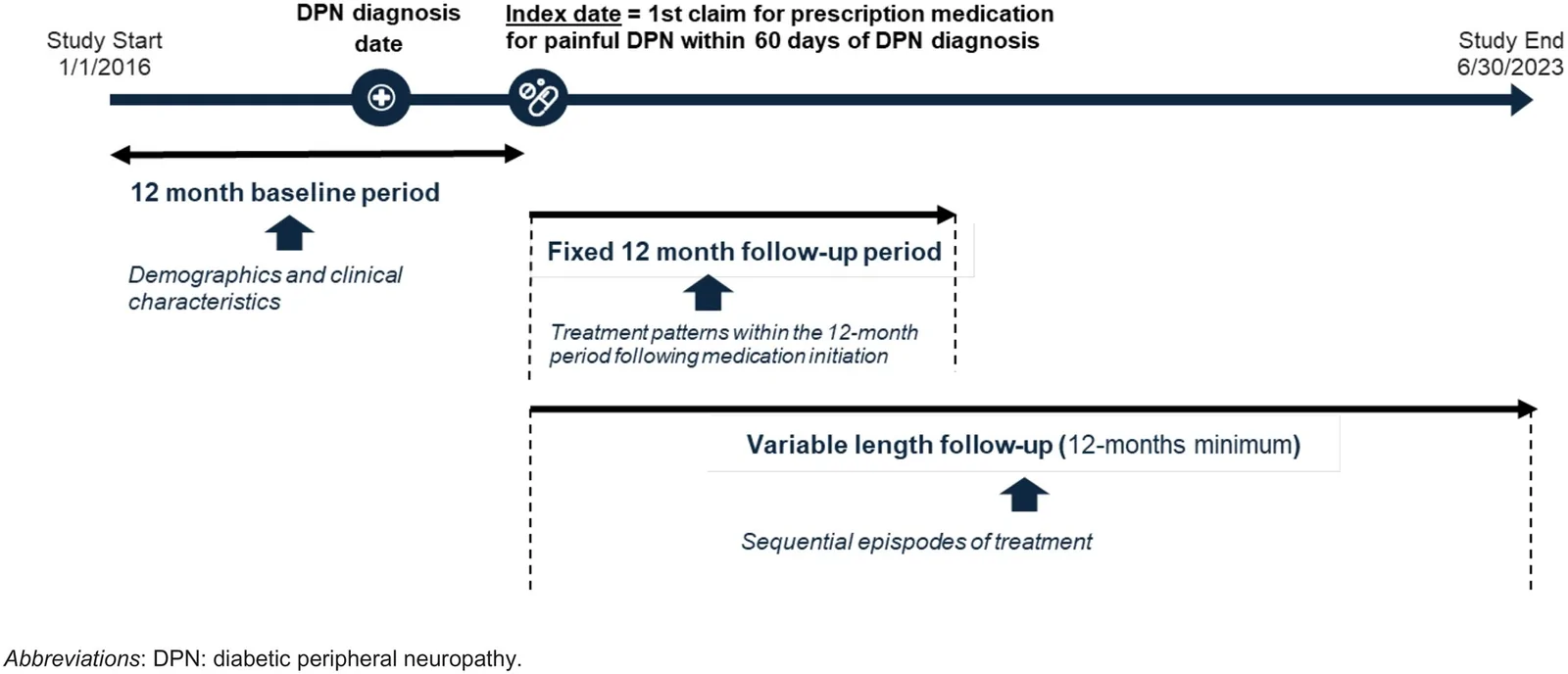
Study design.

This retrospective, observational study was conducted using healthcare claims from the Merative™ MarketScan® Commercial and Medicare Databases from January 1, 2016, to June 30, 2023 (“study period”) to describe treatment patterns among adult patients (aged ≥18 years) with painful DPN who were newly prescribed a pain medication used to treat neuropathic pain; the first prescription date was the index treatment date (Figure 1).

All patients and variables were defined across the databases using International Classification of Diseases, 10th Revision, Clinical Modification (ICD-10-CM) codes, Current Procedural Terminology (CPT) 4th edition codes, Healthcare Common Procedure Coding System (HCPCS) codes, and National Drug Codes (NDC). All database records are de-identified and certified to be fully compliant with the US patient confidentiality requirements set forth in the Health Insurance Portability and Accountability Act of 1996.

### Patient selection and study cohorts

Patients aged 18 years or older who had at least one diagnosis for diabetes mellitus, at least one diagnosis for DPN, and at least one pharmacy or medical claim for a medication used to treat neuropathic pain within 60 days of a DPN diagnosis between January 1, 2017, and June 30, 2022, were included. Pain medications included in the analysis were based on the American Academy of Neurology (AAN) 2021 guidelines for painful DPN, and included gabapentinoids, antidepressants, opioids, sodium channel antagonists, and topical agents (Table S1).^9^ Patients were required to remain continuously enrolled in the database for 12 months prior to the index treatment date (baseline) and for a minimum of 12 months after (follow-up). The follow-up period was variable in length (minimum 12 months) from index treatment date until disenrollment or end of study period.

To increase the likelihood of including patients newly initiating treatment for painful DPN, patients with claims for medications used to treat neuropathic pain during the baseline period were excluded. Given that most pain medications are also approved for indications other than painful DPN, including depression, epilepsy, postherpetic neuralgia, restless leg syndrome, fibromyalgia, low back pain, or painful neuropathy other than DPN (ie, trigeminal nerve disorders, drug induced neuropathy, hereditary neuropathy), patients with documented diagnoses of these conditions within 60 days prior to the index treatment date were excluded to reduce potential confounding. Further, patients with a major surgical procedure in the 30 days prior to or following the index treatment date were excluded to ensure those receiving opioids for the management of post-surgical acute pain were not included. Major surgical procedure was defined using the Healthcare Cost and Utilization Project [HCUP] procedure codes and classifications.^16^ Finally, patients with a diagnosis for malignancy or metastatic disease during the baseline period were excluded to avoid inclusion of patients receiving medication for cancer-related pain.

Subgroups of patients who initiated treatment with FDA approved or commonly used pain medications for painful DPN, specifically duloxetine, gabapentin, pregabalin, and tramadol, were identified to evaluate treatment pattern outcomes among these patients. Other medications (eg, amitriptyline, oxycodone, carbamazepine) were not included in the detailed treatment pattern analysis due to low utilization (Table S2).

### Treatment episodes

For all patients meeting study eligibility criteria, up to four sequential treatment episodes (TEs) were defined in the variable-length follow-up period to characterize treatment patterns (TE1, TE2, TE3, TE4). The start date for the first TE was the index treatment date and all claims for medications used to treat neuropathic pain prescribed on that date defined the treatment regimen, which could be a monotherapy or combination therapy. The end of the TE was defined as the earliest of a discontinuation (defined as a gap of at least 90 days following the end of days’ supply for all treatments in the regimen), switch (claim for another drug not included in the regimen and dependent on the discontinuation of all regimen treatments), or censoring due to end of follow-up (defined as end of study period or disenrollment from the database). The addition of a new pain medication while continuing the TE regimen was evaluated but considered a medication addition and not a new TE. A subsequent TE was defined to start on the date of a switch or on the date of restarting treatment following discontinuation and considered eligible only if there was a DPN diagnosis within 60 days of the start date.

### Outcomes and analyses

#### Demographics and clinical characteristics

Baseline demographics were measured on the index treatment date. Clinical characteristics were assessed during the 12-month baseline period and included the Deyo–Charlson Comorbidity Index (CCI), the Diabetes Complications Severity Index, type of diabetes diagnosis, and DPN-related comorbid conditions.^17^ The duration of the variable length follow-up was also reported.

#### Index treatment and treatment patterns over first 12 months of follow-up

The index treatment regimen type (monotherapy or combination therapy) and pain medication(s) were captured. Discontinuation of index treatment(s) was reported during the initial 12 months of follow-up as well as in 3- or 6-month intervals (0–3 months, 4–6 months, 7–12 months) overall and among the subgroups of patients who indexed with duloxetine, gabapentin, pregabalin, or tramadol.

Dosage, adherence, and treatment alterations of the index treatment were only reported among the subgroups of patients indexing on duloxetine, pregabalin, gabapentin, and tramadol. The average daily dose of the index treatment was calculated as strength multiplied by quantity then divided by days’ supply; change in dose was measured among patients with at least two prescriptions for the index treatment in the first 12 months of follow-up. Adherence was defined as the proportion of days covered (PDC) and calculated using the total days covered by the medication divided by the measurement period (365 days). High adherence was defined as a PDC ≥ 0.8, medium as 0.5 ≤ PDC < 0.8, and low adherence PDC < 0.5.^14^^,^^18^ Treatment alterations in the first 12 months of follow-up were classified into mutually exclusive categories as follows: (1) discontinuation of the index regimen with switch to another medication; (2) discontinuation of the index regimen with no switch to another medication; (3) continuation of the index regimen with addition of another medication; (4) continuation of the index regimen with no addition of another medication.

#### Treatment patterns over variable-length follow-up

The following outcomes were reported for each of the four sequential TEs: Duration of TE, time between TEs, end reason for each TE (switching, discontinuation, censoring [end of study period or end of enrollment]), and addition of another treatment during the TE. Sankey diagrams were used to illustrate the sequence of TE regimens at the patient level; mutually exclusive regimens were defined based on the list if medications used to treat neuropathic pain provided in Table S1.

#### Analyses

Descriptive analyses were conducted for all study measures, overall and for the index treatment subgroups. Categorical variables were presented as the count and percentage of patients in each category, while continuous variables were summarized by providing the mean, median, and standard deviation (SD). Kaplan–Meier (KM) analysis was used to describe time to discontinuation. Sensitivity analyses were conducted to understand the potential impact of the COVID-19 pandemic period by stratifying results by patients with a follow-up period ending before March 1, 2020, and patients with an index date on or after March 1, 2020, through June 30, 2022. All analyses were performed using SAS 9.4 (SAS Institute, Inc, Cary, NC, USA).

## Results

### Study population

**Figure 2:**
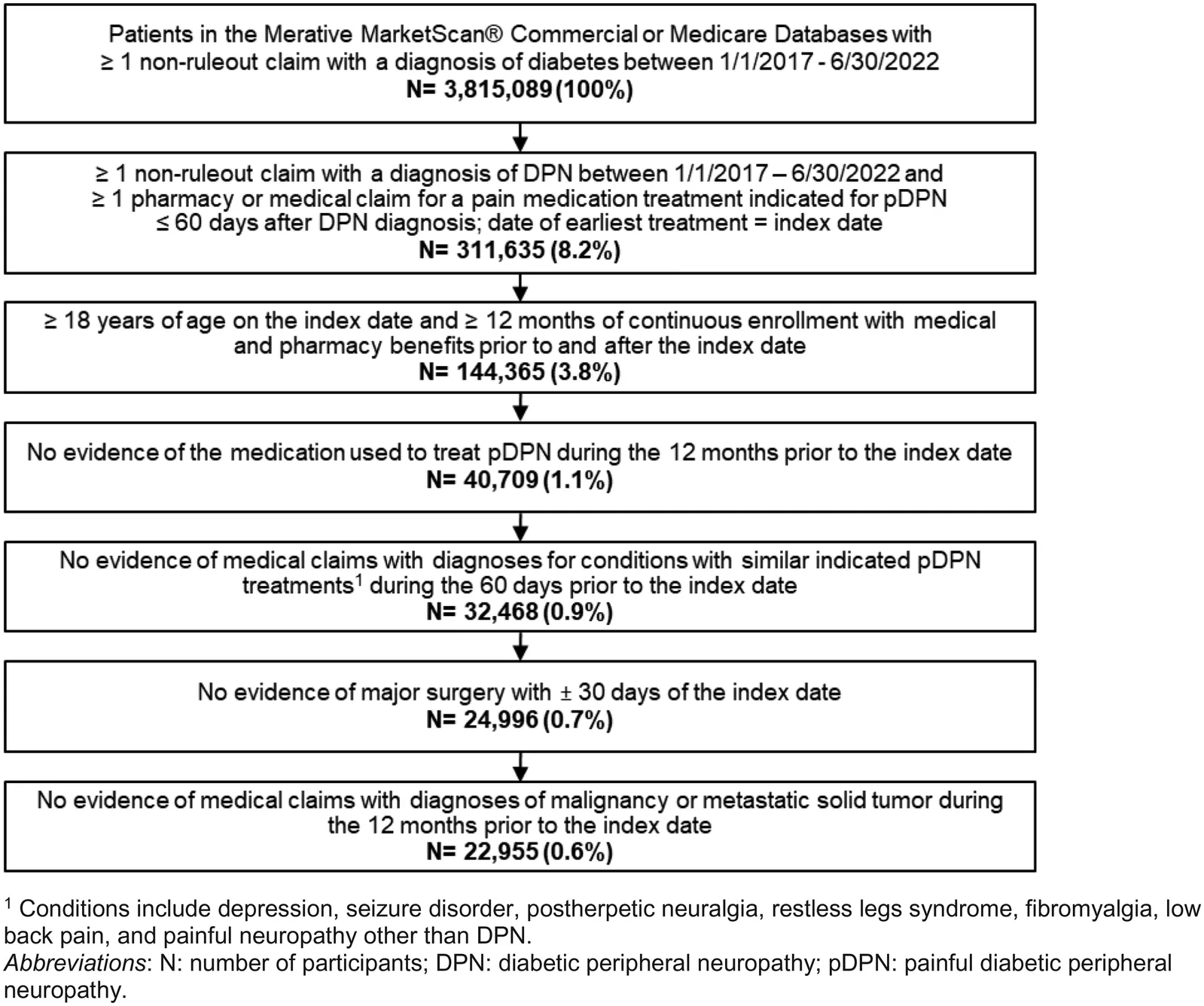
Patient selection.

A total of 22 955 patients met the study inclusion criteria (Figure 2). Patients with DPN newly initiating pain medications had a mean (SD) age of 60.7 (12.8) years, and 55.1% were male (Table 1). The mean (SD) duration of follow-up was 1024 (529) days. Patients had a mean (SD) CCI score of 2.8 (1.6). Hypertension (75.4%), lipid disorders (68.0%), and obesity (36.1%) were the most common comorbid conditions followed by cardiovascular disease (29.0%), nephropathy (26.9%), and sleep disorders (22.3%).

**Table 1:** Demographic and clinical characteristics.

	All patients
	*N* = 22 955
**Demographic characteristics: On index**	
Age, mean (SD)	60.7 (12.8)
Median	60.0
Age category, *N* (%)	
18–29	170 (0.7%)
30–39	794 (3.5%)
40–49	3086 (13.4%)
50–59	7362 (32.1%)
60–69	5760 (25.1%)
70–79	3732 (16.3%)
80–89	1809 (7.9%)
89+	242 (1.1%)
Sex, *N* (%)	
Male	12 648 (55.1%)
Female	10 307 (44.9%)
Geographic region, *N* (%)	
Northeast	2722 (11.9%)
North central	7056 (30.7%)
South	10 654 (46.4%)
West	2481 (10.8%)
Unknown	42 (0.2%)
Insurance plan type, *N* (%)	
Comprehensive/indemnity	2914 (12.7%)
EPO/PPO	11 886 (51.8%)
POS/POS with capitation	1155 (5.0%)
HMO	3561 (15.5%)
CDHP/HDHP	3106 (13.5%)
Other/Unknown	333 (1.5%)
Payer type, *N* (%)	
Commercial	15 429 (67.2%)
Medicare	7526 (32.8%)
Duration of variable-length follow-up in days, mean (SD)	1024 (529)
Median	876
**Clinical characteristics: During 12 month baseline**	
Deyo–Charlson comorbidity index, mean (SD)	2.8 (1.6)
Median	2
Deyo-Charlson comorbidity index, *N* (%)	
0	826 (3.6%)
1	3547 (15.5%)
2	8683 (37.8%)
3	3356 (14.6%)
4+	6543 (28.5%)
Diabetes complications severity index, mean (SD)	2.6 (1.8)
Median	2
Type of diabetes diagnosis (*N*, %)^a^	
Diabetes mellitus due to underlying condition	959 (4.2%)
Drug or chemical induced diabetes mellitus	25 (0.1%)
Type 1 diabetes mellitus	2955 (12.9%)
Type 2 diabetes mellitus	21 647 (94.3%)
Other specified diabetes mellitus	1247 (5.4%)
Painful DPN-related comorbid conditions, *N* (%)	
Hypertension	17 298 (75.4%)
Lipid disorders (ie, hyperlipidemia)	15 602 (68.0%)
Obesity	8283 (36.1%)
Cardiovascular disease	6661 (29.0%)
Nephropathy	6179 (26.9%)
Sleep disorders	5119 (22.3%)
Diabetes-related infections^b^	4493 (19.6%)
Anxiety	2205 (9.6%)
Depression	1300 (5.7%)
Vitreous detachment	893 (3.9%)
Cognitive dysfunction	630 (2.7%)
Hypoglycaemia	379 (1.7%)

a Categories were not mutually exclusive.

b Diabetes-related infections include respiratory infections, urinary tract infections, gastrointestinal and liver infections, soft tissue and bone infections, head and neck infections.

Abbreviations: DPN, Diabetic peripheral neuropathy; EPO, Exclusive provider organization; HMO, Health maintenance organization; N, Number of participants; POS, Point of service; PPO, Preferred provider organization; CDHP, Consumer-driven health plan; HDHP, High deductible health plan; SD, Standard deviation.

### Index treatment and treatment patterns over first 12 months of follow-up

#### Index treatment

Among the 22 955 patients identified, 98.5% (*n* = 22 616) indexed on a monotherapy treatment for painful DPN and 1.5% (*n* = 339) indexed on a combination therapy (Table 2). The majority of patients indexed on gabapentinoids (gabapentin 59.0%; pregabalin 5.3%), opioids (tramadol 15.1%, oxycodone 7.0%), or antidepressants (duloxetine 5.2%, amitriptyline 2.4%). Among the 1.5% of patients who indexed on ≥2 medications, most patients initiated with gabapentin combined with either tramadol, oxycodone, duloxetine, lidocaine, or amitriptyline (Table S2).

**Table 2:** Index treatments.

	All patients
	*N* = 22 955
**Patients on a monotherapy index regimen, *N* (%)**	**22** **616 (98.52%)**
**Patients on a combination index regimen, *N* (%)**	**339 (1.48%)**
**Treatment utilization at index, *N (%)*** *^a^*	
**Gabapentinoids**	**14** **749 (64.25%)**
Gabapentin	13 533 (58.95%)
Pregabalin	1216 (5.30%)
**SNRI antidepressants**	**1476 (6.43%)**
Desvenlafaxine	31 (0.14%)
Duloxetine	1196 (5.21%)
Venlafaxine	249 (1.08%)
**Tricyclic antidepressants**	**852 (3.71%)**
Amitriptyline	554 (2.41%)
Desipramine	8 (0.03%)
Imipramine	12 (0.05%)
Nortriptyline	278 (1.21%)
**Opioids**	**5108 (22.25%)**
Dextromethorphan	6 (0.03%)
Morphine	35 (0.15%)
Oxycodone	1595 (6.95%)
Tramadol	3462 (15.08%)
Tapentadol	10 (0.04%)
**Topical agents**	**986 (4.30%)**
Capsaicin	4 (0.02%)
Lidocaine	982 (4.28%)

a Drug classes with >1% of patients were reported; <1% of patients initiated on sodium channel antagonists and no patients indexed on valproate.

Abbreviations: N, Number of participants; SD, Standard deviation; SNRI, Serotonin and norepinephrine reuptake inhibitors.

#### Prescribed doses of index treatments

**Figure 3:**
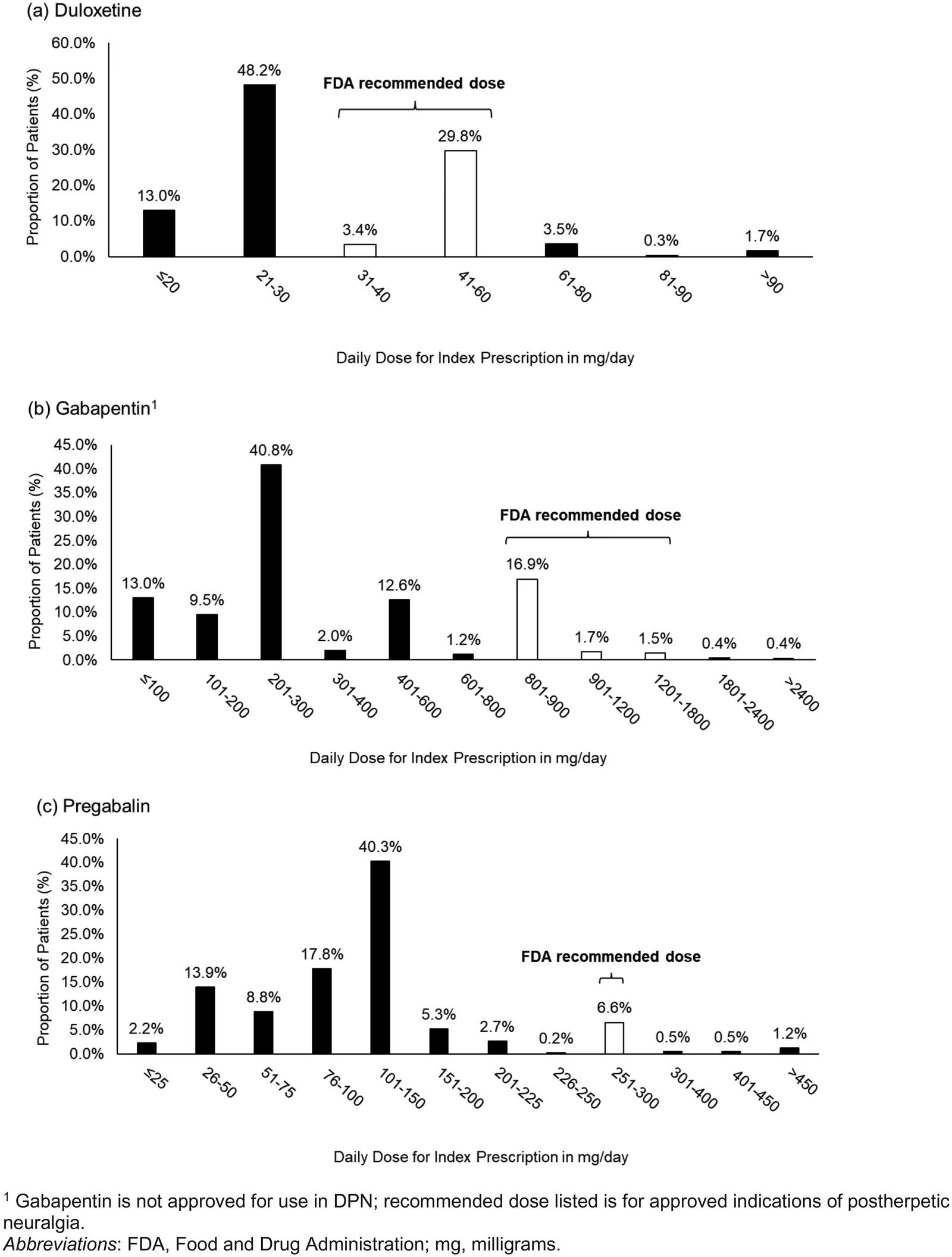
Average daily dose of the index medication (A) Duloxetine; (B) Gabapentin; (C) Pregabalin.

The distribution of the average daily dose of the first prescription for duloxetine, gabapentin, and pregabalin are shown in Figure 3. The majority of patients indexing on pregabalin (91.2%) or duloxetine (61.3%) received doses below the FDA-recommended levels for the treatment of painful DPN (300 mg/day for pregabalin and 40–60 mg/day for duloxetine).^19^^,^^20^ Approximately 80% of patients indexing on gabapentin were prescribed doses below the FDA-recommended daily therapeutic range (900–1800 mg/day) for the FDA-approved indication of postherpetic neuralgia.^21^ Among patients indexing on gabapentin with at least two prescription fills, only 14.1% increased to a higher dose, while the majority (81.0%) maintained their initial dosage (Table 3). Similarly, dose escalation was observed in just 5.3% of patients treated with pregabalin and 2.5% with duloxetine, with most maintaining their initial dose (89.5% for pregabalin and 96.4% for duloxetine). Among patients indexing on tramadol, 65.6% had evidence of only one prescription; and among the few with at least two prescription fills, most (83.7%) maintained their initial dose, which was a median dose of 200 mg/day, consistent with FDA recommendations (Table 3).^22^

**Table 3:** Dose, adherence, and discontinuation over first 12 months follow-up by index treatment subgroups.

	Duloxetine	Gabapentin	Pregabalin	Tramadol
	*N* = 1196	*N* = 13 533	*N* = 1216	*N* = 3462
**Dose**				
Average dose per day, first prescription (median)	30.0	300.0	150.0	200.0
Patients with at least 2 prescriptions, *N* (%)	855 (71.5%)	9852 (72.8%)	796 (65.5%)	1190 (34.4%)
Dose increase	21 (2.5%)	1392 (14.1%)	42 (5.3%)	82 (6.9%)
Dose decrease	11 (1.3%)	482 (4.9%)	42 (5.3%)	112 (9.4%)
No dose change	823 (96.3%)	7978 (81.0%)	712 (89.5%)	996 (83.7%)
**Adherence**				
Proportion of days covered (PDC), mean (SD)	0.5 (0.4)	0.5 (0.3)	0.4 (0.3)	0.1 (0.1)
Adherence, *N* (%)				
High adherence (PDC ≥ 0.80)	449 (37.5%)	3622 (26.8%)	232 (19.1%)	30 (0.9%)
Medium adherence (0.5≤ PDC < 0.8)	113 (9.5%)	1916 (14.2%)	154 (12.7%)	54 (1.6%)
Low adherence (PDC < 0.5)	634 (53.0%)	7995 (59.1%)	830 (68.3%)	3378 (97.6%)
**Discontinuation**				
Patients with discontinuation, *N* (%)	763 (63.8%)	9462 (69.9%)	906 (74.5%)	3350 (96.8%)
Discontinue during months 0–3	482 (40.3%)	6052 (44.7%)	623 (51.2%)	3059 (88.4%)
Discontinue during months 4–6	132 (11.0%)	1685 (12.5%)	151 (12.4%)	194 (5.6%)
Discontinue during months 7–12	149 (12.5%)	1725 (12.7%)	132 (10.9%)	97 (2.8%)
Days to discontinuation, mean (SD)	105.4 (90.5)	101.1 (88.6)	86.5 (80.1)	27.0 (50.7)
Median	90.0	75.0	47.5	7.0

Abbreviations: *N*, Number of participants; PDC, Proportion of days covered; SD, Standard deviation.

#### Adherence

Low adherence was observed across all index treatment groups, with a mean PDC of ≤0.5 during the first 12 months following initiation (Table 3). More than half of the patients indexing on duloxetine, gabapentin, or pregabalin demonstrated low adherence (53.0%, 59.1%, and 68.3% respectively), while nearly all patients treated with tramadol demonstrated low adherence (97.6%).

#### Discontinuation

**Figure 4:**
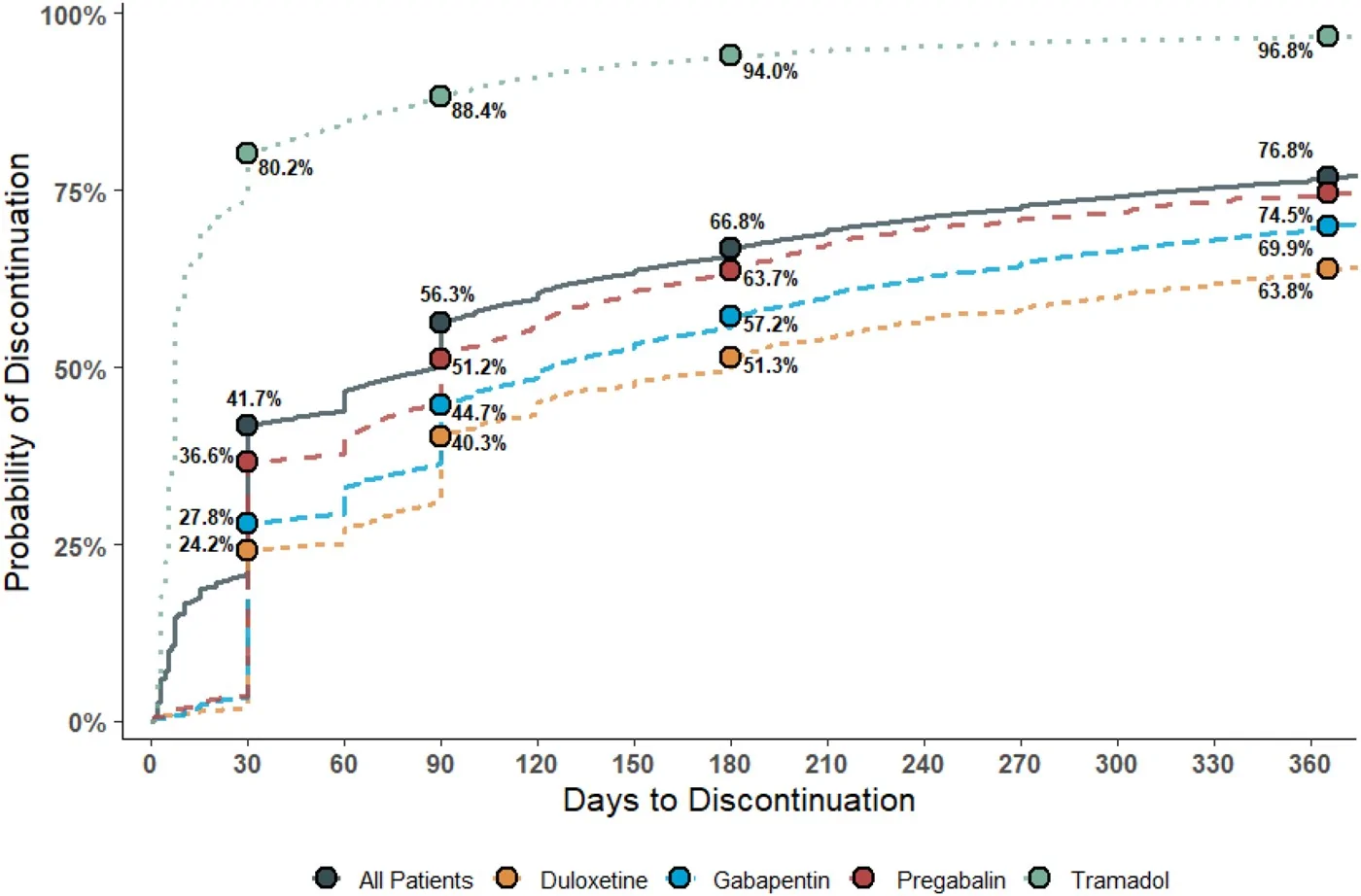
Kaplan–Meier time to discontinuation over first 12 months follow-up among all medications and index treatment subgroups.

Over half (56.3%) of all patients discontinued their index treatment within 3 months of initiation, increasing to almost 77% in the first 12 months (Figure 4). The 1-year discontinuation rates for the most commonly used drug class to the least were gabapentin (69.9%), tramadol (96.8%), pregabalin (74.5%), and duloxetine (63.8%). The median time to discontinuation was 75.0 days for patients indexing on gabapentin, 7.0 days for tramadol, 47.5 days for pregabalin, and 90.0 days for duloxetine.

#### Treatment alterations over first 12 months of follow-up

**Figure 5:**
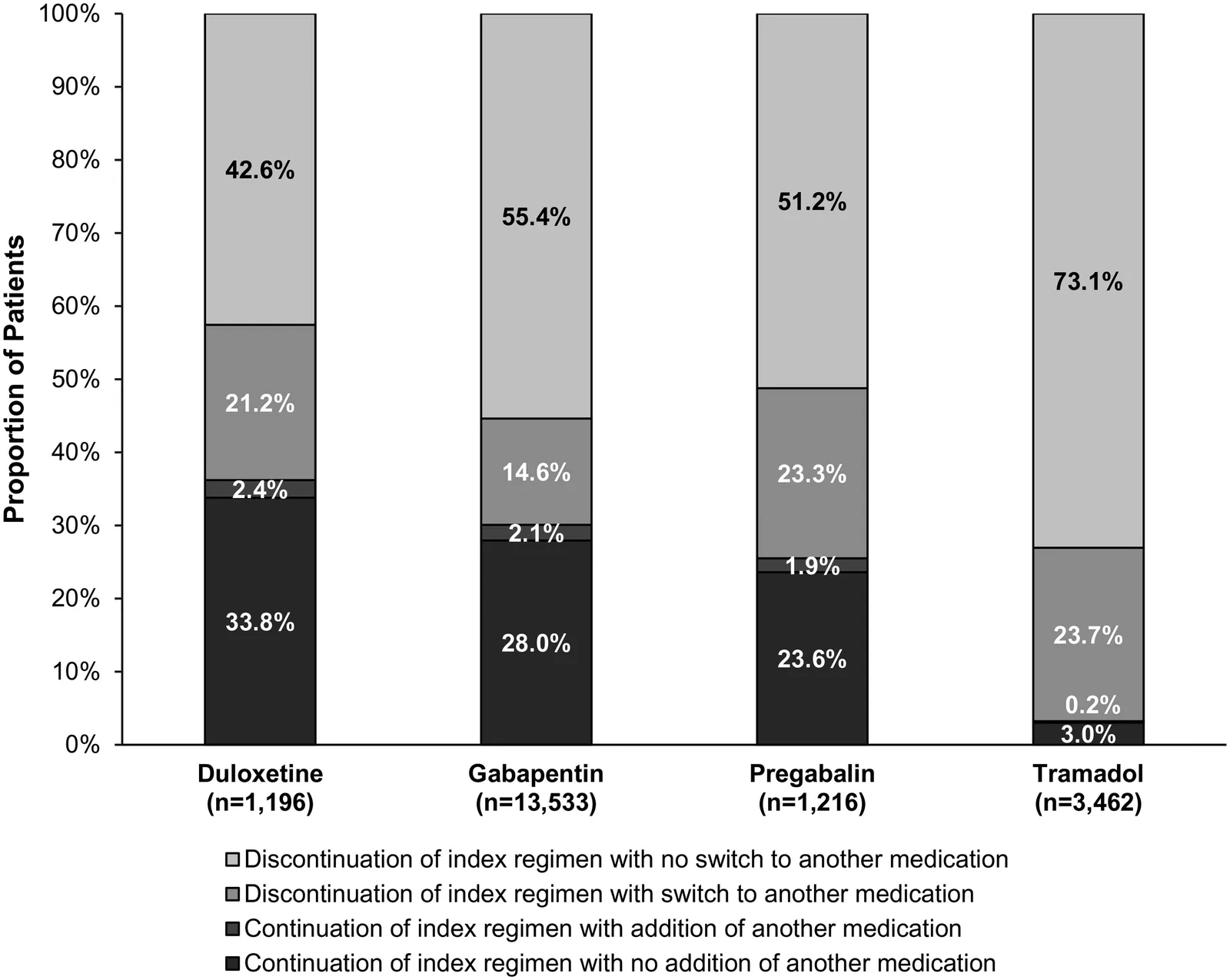
Treatment alterations over first 12 months follow-up by index treatment subgroups.

Despite high discontinuation rates of index treatment, the greatest proportion of patients discontinued treatment without switching to or adding another pain medication within 12 months of follow-up (Figure 5). Fewer than 25% of patients transitioned to an alternative medication to manage neuropathic pain after discontinuation. Among those who did switch or restart treatment, most moved to another drug within the same class or a medication from a different class. Approximately 2% of patients added a new pain medication while remaining on their index treatment in the 12 months of follow-up. The most common additions were opioids (oxycodone and tramadol) for all patients and gabapentin specifically for those indexing on duloxetine or tramadol.

### Treatment patterns over variable-length follow-up

**Figure 6:**
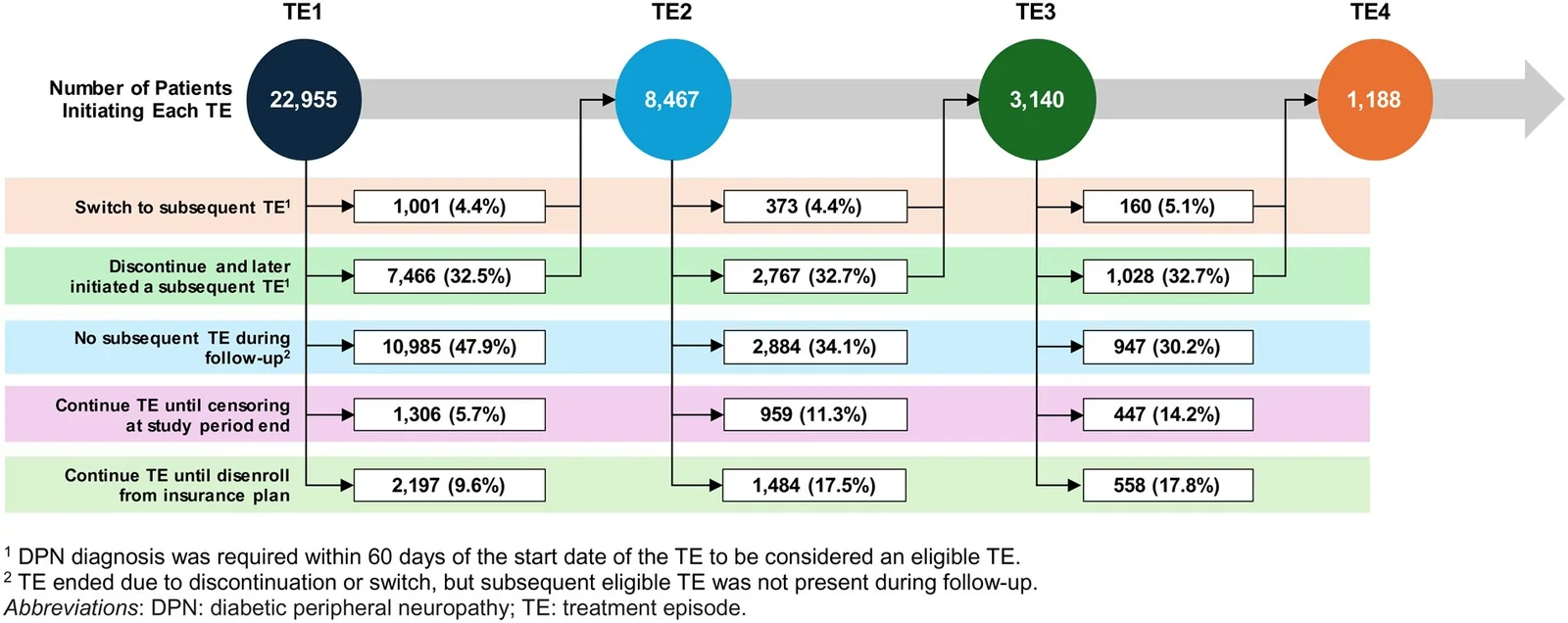
Treatment episodes over variable-length follow-up.

Of the 22 955 patients newly initiating treatment to manage painful DPN, 36.9% (*N* = 8467) moved on to TE2, 13.7% (*N* = 3140) to TE3, and 5.2% (*N* = 1188) to TE4 during the variable length follow-up period (Table 4, Figure 6). Less than 10% of patients switched treatment and moved onto a subsequent TE. Across the 4 TE’s, approximately one third of patients (32.5% TE1, 32.7% TE2, 32.7% TE3) discontinued their TE (gap ≥90 days) and later initiated a subsequent TE after a gap, of which nearly half restarted on a different regimen. Almost 48% (*N* = 10 985) of patients did not initiate a subsequent TE after discontinuing TE1. Cumulatively, nearly 65% of patients discontinued and did not have a subsequent TE across the four TEs (10 985 discontinued after TE1, 2884 discontinued after TE2, and 947 discontinued after TE3).

**Table 4:** Treatment episodes over variable-length follow-up.

	TE1	TE2	TE3	TE4
	*N* = 22 955	*N* = 8467	*N* = 3140	*N* = 1188
TE end reason, *N* (%)^a^				
**Switching**	**1160 (5.1%)**	**412 (4.9%)**	**172 (5.5%)**	**78 (6.6%)**
and moved on to a subsequent pDPN TE^b^	1001 (4.4%)	373 (4.4%)	160 (5.1%)	
and did not move on to a subsequent pDPN TE^b^	159 (0.7%)	39 (0.5%)	12 (0.4%)	
**Discontinuation**	**18** **292 (79.7%)**	**5612 (66.3%)**	**1963 (62.5%)**	**715 (60.2%)**
and moved on to a subsequent pDPN TE^b^	7466 (32.5%)	2767 (32.7%)	1028 (32.7%)	
restarted on same regimen	3817 (16.6%)	1294 (15.3%)	468 (14.9%)	
restarted on different regimen	3649 (15.9%)	1473 (17.4%)	560 (17.8%)	
and did not move on to a subsequent pDPN TE^b^	10 826 (47.2%)	2845 (33.6%)	935 (29.8%)	
**Censoring**	**3503 (15.3%)**	**2443 (28.9%)**	**1005 (32.0%)**	**395 (33.3%)**
End of study period (6/30/2023)	1306 (5.7%)	959 (11.3%)	447 (14.2%)	198 (16.7%)
End of enrollment	2197 (9.6%)	1484 (17.5%)	558 (17.8%)	197 (16.6%)
**Duration of TE** ^a^ **in days, mean (SD)**	**239 (368)**	**188 (281)**	**171 (257)**	**154 (226)**
Median	84	90	84	79
25th, 75th percentile	30, 315	30, 229	30, 204	29, 180
**Time between TEs in days, mean (SD)**		**328 (353)**	**258 (282)**	**202 (218)**
Median		210	168	139
25th and 75th percentile		97, 442	76, 340	54, 271
**Added-on a new treatment during TE, *N* (%)**	**1307 (5.7%)**	**495 (5.8%)**	**209 (6.7%)**	**73 (6.1%)**

a TE ends at the earlier of discontinuation (90+ day gap), switch to another drug different than index, or censoring at end of study period.

b DPN diagnosis was required within 60 days of the switched drug to be considered a valid TE.

Abbreviations: *N*, Number of participants; pDPN, Painful diabetic peripheral neuropathy; SD, Standard deviation; TE, Treatment episode.

The median duration of each TE was approximately 3 months (Table 4). The median time between TEs (ie, time off treatment from prior TE end to next TE start) decreased in subsequent episodes from 210 days between TE1 and TE2 to 139 days between TE3 and TE4. The addition of another medication during each TE was infrequent (5%–7%).

**Figure 7:**
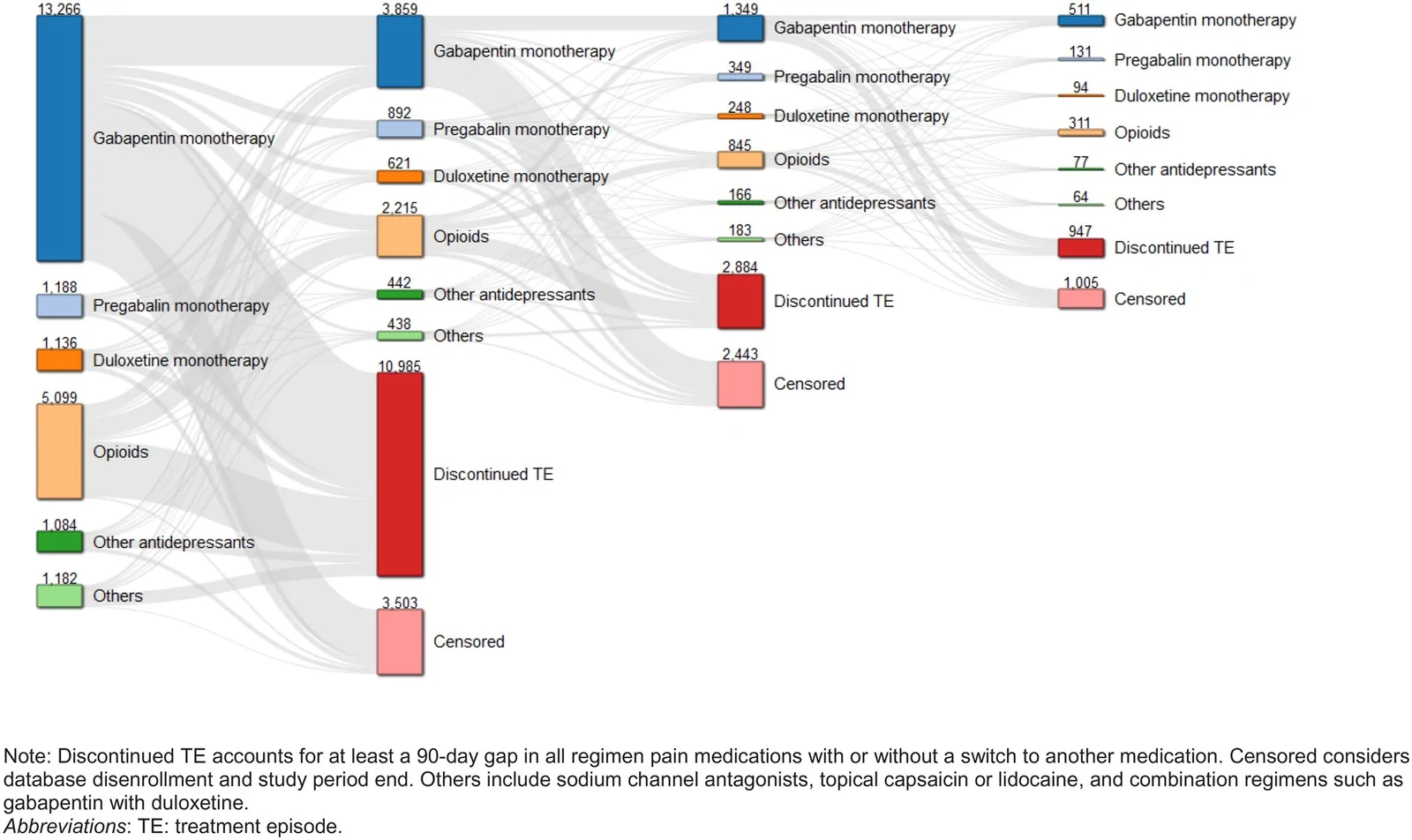
Sankey diagram depicting treatment sequencing through four treatment episodes over variable-length follow-up.

When following patients longitudinally through their treatment journey, gabapentin monotherapy was the most common regimen across TEs; the proportion of patients treated with gabapentin decreased across TEs (from 57.8% in TE1, 45.6% in TE2, 43.0% in TE3, 43.0% in TE4) (Figure 7, Table S2). Patients treated with pregabalin monotherapy increased across TEs from 5.2% in TE1 to 11.0% in TE4, as did patients treated with duloxetine monotherapy from 4.9% in TE1 to 7.9% in TE4. Opioid use remained consistent across TEs (22.2%–26.9%).

### Sensitivity analysis

Similar results were observed between the COVID-19 pandemic and pre-COVID time periods as detailed in the Supplement (Table S3).

## Discussion

This study found that gabapentinoids (gabapentin 59.0%; pregabalin 5.3%), opioids (tramadol 15.1%, oxycodone 7.0%), and antidepressants (duloxetine 5.2%, amitriptyline 2.4%) were the most frequently prescribed medications for patients newly initiating treatment for painful DPN. The majority were prescribed daily doses below the FDA-recommended levels for gabapentin (79%), pregabalin (91%), and duloxetine (61%), with most (81%–96%) maintaining their initial subtherapeutic dosage. Treatment adherence was suboptimal among patients initiating gabapentin, pregabalin, or duloxetine (mean PDC ≤ 0.5); approximately 75% discontinued therapy within 1-year (approximately 50% discontinued within the first 3 months), and fewer than 25% switched to a new medication after discontinuation. Furthermore, when evaluating treatment patterns across four TEs, almost 65% discontinued all neuropathic pain medications and did not receive subsequent treatment by the last TE.

Findings from this study build upon and update prior research, which evaluated treatment patterns among patients with painful DPN within a fixed 12-month follow up of initiating treatment using datasets from 2006 to 2011.^14^ When comparing the results of this study with these previously published findings, the proportion of patients initiating treatment with pregabalin decreased by over 75% (21.6% to 5.3%) while the proportion initiating treatment with gabapentin increased by nearly 30% (45.0% to 59.0%). Furthermore, while treatment initiation with duloxetine remained consistent (5.2%), the proportion initiating with TCAs decreased by nearly 70% (11.3% to 3.7%) and the proportion initiating with opioids increased by 75% (12.7% to 22.3%) between the 2 studies. While patient demographic characteristics were similar, some clinical characteristics differed, likely due to varying coding definitions.^14^ Diabetes-related infections, nephropathy, and obesity, while more prevalent in the current study sample, were as expected and consistent with other published studies given the interrelation of these conditions with diabetes.^23–26^

Although widely prescribed, real-world use of current pharmacotherapies for managing painful DPN often do not provide adequate pain relief and may cause adverse events that reduce tolerability, frequently resulting in discontinuation of the medication.^27^ In this study, gabapentin was the most frequently prescribed initial treatment, despite its off-label use for treating painful DPN. Studies have shown that while gabapentin may reduce neuropathic pain for some patients, its analgesic effect is often modest and not universally effective.^28^ Reports indicate that more than half of individuals treated with gabapentin in real-world setting experience adverse effects without adequate pain relief.^29^ Common adverse events associated with gabapentin, such as dizziness, somnolence, fatigue, peripheral edema, and cognitive disturbances, may limit dose escalation and patient adherence.^30^

Despite receiving FDA approval for the treatment of painful DPN more than 20 years ago, first-line utilization rates for pregabalin and duloxetine observed in this study were low (∼5%).^31–34^ The limited prescribing of duloxetine and high first-line prescribing of gabapentin by healthcare providers is surprising given that gabapentin is used off-label for painful DPN, the dosing schedule is more complex, and findings from this study demonstrate higher rates of adherence and lower rates of discontinuation for patients initiating treatment with duloxetine (vs gabapentin). The low prescribing rate of antidepressants, specifically duloxetine, other SNRIs, and TCAs, may be attributable to common side effects such as nausea, dizziness, somnolence/drowsiness, fatigue, dry mouth, constipation, and orthostatic hypotension.^35^^,^^36^ In addition, patients may be reluctant to take SNRIs or TCAs for painful DPN due to the stigma of antidepressants or concerns regarding serotonin syndrome, mood changes, or suicidality.^37^ The low prescribing rate of pregabalin may be attributable to common side effects (such as sedation, dizziness, peripheral edema, weight gain), a perception of limited incremental benefit (vs gabapentin), and its status as a Schedule V controlled substance, which is associated with abuse and safety concerns.^19^

In this study, most patients were prescribed initial doses of duloxetine, gabapentin, or pregabalin at doses below recommended therapeutic levels, with only a small proportion of patients increasing their dosage. While this may reflect concerns about safety and tolerability, it likely contributes to suboptimal treatment outcomes. Notably, more than 80% of patients prescribed pregabalin received ≤150 mg/day, doses that have not demonstrated significant benefit over placebo.^38^^,^^39^ Similarly, nearly two-thirds of patients received duloxetine at doses ≤40 mg/day and were not titrated up over 12 months of follow-up, despite a lack of evidence supporting clinical efficacy at such low levels.^40^

Nearly a quarter of patients with painful DPN were prescribed opioids (most commonly tramadol) as the initial treatment. The proportion of patients with opioid prescriptions was consistent across TEs (22.2%–26.9%) and is a cause for potential concern given opioids can lead to opioid use disorder, and also carry risk of respiratory depression.^41^ Among patients who initiated treatment with tramadol, 97% discontinued therapy, with at least 50% within 7 days, and 98% had low adherence, indicating short-term use consistent with clinical guidelines for managing breakthrough pain.^9^^,^^12^ Tapentadol extended release, the only opioid approved for the treatment of painful DPN, was rarely utilized in this study (<1%).^42^^,^^43^

According to the AAN 2021 guidelines and published evidence-based treatment strategies, up to 12-weeks are recommended to adequately assess the efficacy of medications for neuropathic pain.^9^^,^^27^ However, in this study, 60% of patients discontinued treatment within 3 months of treatment initiation, and 77% discontinued within 1 year. Since adverse events are most likely to occur early in the treatment course, poor tolerability may contribute to these high discontinuation rates.^9^^,^^44^ These findings are consistent with those of Yang et al., who reported that only about one-quarter of patients with painful DPN maintain on a stable treatment regimen during the first year.^14^ Similarly, the low adherence (PDC ≤ 0.5) observed in both studies suggests inadequate treatment outcomes and poor pain control.

Despite high discontinuation rates, most patients were not prescribed a different pain medication. Across the 4 TEs assessed over the variable follow-up period, only 5%–7% of patients switched medications before discontinuation, and just 16%–18% initiated a new regimen after stopping treatment. Among those who did transition to a subsequent TE, the median time between TEs exceeded 3 months, resulting in treatment interruptions without pharmacologic pain management.

Several factors may explain delays in starting or continuing treatment for neuropathic pain. The medications are often associated with side effects such as sedation, dizziness, cognitive impairment, or weight gain.^45^^,^^46^ In addition, subtherapeutic dosing or inadequate analgesic response may cause patients to lose confidence in treatments. The burden of polypharmacy in diabetes management may also reduce willingness among both providers and patients to consider adding another medication (particularly one with suboptimal effectiveness), especially when pain is intermittent or fluctuating in intensity.^46^ Furthermore, inadequate guidance due to limited access to specialists and inconsistent follow-up may delay treatment adjustments.^47^ Although published guidelines, evidence-based treatment strategies, and various provider education resources on therapeutic doses are readily available, real-world data reveal that pain relief with current oral pharmacotherapies for painful DPN remains insufficient, highlighting a significant unmet need among these patients for novel pharmacotherapies.^9^^,^^27^^,^^48^ These findings underscore the complexity of managing painful DPN and highlight the importance of better treatment options.

### Limitations

The results of this study should be examined while considering their limitations, many of which are inherent to retrospective study design and use of claims data. First, patients were identified using a coding algorithm due to the absence of ICD-10-CM diagnosis codes for painful DPN, which may have artificially restricted the sample but were necessary to ensure a DPN patient population newly initiating therapy for the management of pain. The criteria of pain medications used to treat neuropathic pain within 60 days of a DPN diagnosis was necessary to ensure the medication was for pain associated with DPN, even though patients may be excluded if their pain medication was prescribed beyond the 60-day window. Additionally, the exclusion diagnoses were designed to isolate patients with painful DPN, but patients with both painful DPN and comorbid conditions may be excluded. Second, patients were identified as newly treated based on the absence of claims for neuropathic pain medication in the 12 months prior, but medication outside this window may have occurred. Pertinent to capturing treatments, actual usage of filled prescriptions and use of over-the-counter medications cannot be ascertained from healthcare claims. Finally, the dataset included only insured beneficiaries within commercial and Medicare eligible populations, which may limit the generalizability of results to the Medicaid, Veterans Affairs, Department of Defense, and uninsured populations.

## Conclusions

This study provides valuable insights into current real-world treatment patterns among patients with painful diabetic peripheral neuropathy (DPN) in the United States, using a contemporary dataset. The high rates of treatment discontinuation and suboptimal adherence observed in this study suggest challenges with tolerability and limited real-world effectiveness of currently available therapies, often leaving patients with inadequately managed pain. These findings underscore the limitations of existing treatment options and highlight the unmet need for more effective and better-tolerated therapies to address the significant challenges faced by patients with painful DPN.

## Supplementary Material

pnag055_Supplementary_Data

## Data Availability

The data that support the findings of this study are available from Merative. Restrictions apply to the availability of these data, which were used under license for this study.
