# Supplementary material for: Real world treatment patterns among patients with painful diabetic peripheral neuropathy in the United States
Source: Pain Med. 2026 Apr 21;27(9):1027–39. doi: 10.1093/pm/pnag055 (PMC13537738; doi:10.1093/pm/pnag055)
Supplement: Supplementary file 1 [file pnag055_supplementary_data.docx]

**Supplemental Table 1. Neuropathic Pain Medications**

| **Medication Class** | **Generic Name** |
| --- | --- |
| Gabapentinoids | gabapentin |
| Gabapentinoids | pregabalin |
| Serotonin and norepinephrine reuptake inhibitors | desvenlafaxine |
| Serotonin and norepinephrine reuptake inhibitors | duloxetine |
| Serotonin and norepinephrine reuptake inhibitors | venlafaxine |
| Tricyclic Antidepressants | amitriptyline |
| Tricyclic Antidepressants | desipramine |
| Tricyclic Antidepressants | imipramine |
| Tricyclic Antidepressants | nortriptyline |
| Opioids | dextromethorphan |
| Opioids | morphine |
| Opioids | oxycodone |
| Opioids | tapentadol |
| Opioids | tramadol |
| Sodium channel antagonists | valproate |
| Sodium channel antagonists | carbamazepine |
| Sodium channel antagonists | oxcarbazepine |
| Sodium channel antagonists | lamotrigine |
| Sodium channel antagonists | lacosamide |
| N-methyl-D-aspartate (NMDA) antagonist | dextromethorphan |
| Topical analgesic | topical capsaicin |
| Local anesthetic | topical lidocaine |

**Supplemental Table 2. Regimen Distributions by Drug Type for Monotherapies and Combination Therapies**

|  | **TE 1** | **TE 2** | **TE 3** | **TE 4** |
| --- | --- | --- | --- | --- |
|  | **N = 22,955** | **N = 8,467** | **N = 3,140** | **N = 1,188** |
| **Monotherapy (N, %)** | **22,616 (98.5%)** | **8,302 (98.1%)** | **3,058 (97.4%)** | **1,162 (97.8%)** |
| Gabapentin | 13,266 (57.8%) | 3,859 (45.6%) | 1,349 (43.0%) | 511 (43.0%) |
| Tramadol | 3,330 (14.5%) | 1,155 (13.6%) | 421 (13.4%) | 171 (14.4%) |
| Oxycodone | 1,519 (6.6%) | 914 (10.8%) | 358 (11.4%) | 126 (10.6%) |
| Pregabalin | 1,188 (5.2%) | 892 (10.5%) | 349 (11.1%) | 131 (11.0%) |
| Duloxetine | 1,136 (4.9%) | 621 (7.3%) | 248 (7.9%) | 94 (7.9%) |
| Lidocaine | 916 (4.0%) | 295 (3.5%) | 104 (3.3%) | 33 (2.8%) |
| Amitriptyline | 530 (2.3%) | 234 (2.8%) | 75 (2.4%) | 40 (3.4%) |
| Nortriptyline | 268 (1.2%) | 89 (1.1%) | 40 (1.3%) | 19 (1.6%) |
| Venlafaxine | 236 (1.0%) | 94 (1.1%) | 42 (1.3%) | 13 (1.1%) |
| Lamotrigine | 58 (0.3%) | 32 (0.4%) | 19 (0.6%) | 4 (0.3%) |
| Carbamazepine | 35 (0.2%) | 25 (0.3%) | 11 (0.4%) | 7 (0.6%) |
| Oxcarbazepine | 31 (0.1%) | 21 (0.2%) | 7 (0.2%) | 3 (0.3%) |
| Desvenlafaxine | 30 (0.1%) | 17 (0.2%) | 6 (0.2%) | 3 (0.3%) |
| Morphine | 30 (0.1%) | 22 (0.3%) | 16 (0.5%) | 4 (0.3%) |
| Imipramine | 12 (0.1%) | 5 (0.1%) | 2 (0.1%) | 0 (0.0%) |
| Tapentadol | 10 (0.0%) | 7 (0.1%) | 3 (0.1%) | 2 (0.2%) |
| Desipramine | 8 (0.0%) | 3 (0.0%) | 1 (0.0%) | 1 (0.1%) |
| Dextromethorphan | 6 (0.0%) | 3 (0.0%) | 1 (0.0%) | 0 (0.0%) |
| Capsaicin | 4 (0.0%) | 6 (0.1%) | 3 (0.1%) | 0 (0.0%) |
| Lacosamide | 3 (0.0%) | 8 (0.1%) | 3 (0.1%) | 0 (0.0%) |
| **Combination Therapy (N, %)** | **339 (1.5%)** | **165 (1.9%)** | **82 (2.6%)** | **26 (2.2%)** |
| Gabapentin + Tramadol | 93 (27.4%) | 37 (22.4%) | 8 (9.8%) | 1 (3.8%) |
| Gabapentin + Oxycodone | 48 (14.2%) | 26 (15.8%) | 16 (19.5%) | 1 (3.8%) |
| Duloxetine + Gabapentin | 44 (13.0%) | 20 (12.1%) | 10 (12.2%) | 5 (19.2%) |
| Gabapentin + Lidocaine | 31 (9.1%) | 5 (3.0%) | 5 (6.1%) | 1 (3.8%) |
| Amitriptyline + Gabapentin | 16 (4.7%) | 3 (1.8%) | 4 (4.9%) | 1 (3.8%) |
| Lidocaine + Tramadol | 13 (3.8%) | 3 (1.8%) | 2 (2.4%) | 0 (0.0%) |
| Gabapentin + Venlafaxine | 12 (3.5%) | 1 (0.6%) | 2 (2.4%) | 1 (3.8%) |
| Lidocaine + Oxycodone | 10 (3.0%) | 3 (1.8%) | 0 (0.0%) | 0 (0.0%) |
| Gabapentin + Nortriptyline | 8 (2.4%) | 1 (0.6%) | 2 (2.4%) | 1 (3.8%) |
| Pregabalin + Tramadol | 7 (2.1%) | 7 (4.2%) | 0 (0.0%) | 0 (0.0%) |

*Abbreviations*: TE: treatment episode.

**Sensitivity Analysis**

Among the overall population (N=22,955), 11,652 patients (50.8%) were classified as pre-COVID, defined as having completed their 12-month follow up prior to March 1, 2020. A total of 7,667 patients (33.4%) were classified as COVID, defined by an index date between March 1, 2020 and June 30, 2022. The remaining 3,636 patients (15.8%) did not meet criteria for either subgroup. Demographic and clinical characteristics were similar between groups, with slight differences in prevalence of anxiety (8.8% in pre-COVID vs 10.7% COVID), diabetes-related infections (20.8% vs 17.6%, respectively), and obesity (34.0% vs 38.2%, respectively) (Supplemental Table 3). Index treatment utilization of gabapentin (57.7% pre-COVID and 62.2% COVID) and pregabalin (4.7% pre-COVID and 6.0% COVID) showed some increase, while the utilization of opioids decreased (24.2% pre-COVID and 19.0% COVID) slightly, primarily driven by lower tramadol use in the cohort indexing treatment during the COVID timeframe. Discontinuation within 12 months of follow-up was 77% for both groups, but mean time to discontinuation was slightly less for pre-COVID (74 days) compared to COVID (80 days).

Patient characteristics and treatment patterns remained consistent through the COVID-19 pandemic timeframe. The slight differences observed, such as the increase in prevalence of anxiety between pre-COVID and COVID, align with published trends.[1] However, changes in health care utilization and reduced patient-physician interactions during the COVID-19 era may also artificially reduce the observation of relevant outcomes, as may be the case for diabetes-related infections.

**Supplemental Table 3. Sensitivity Analysis by timing of COVID-19 pandemic period**

|  | **Pre-COVID^1^** | **COVID^1^** |
| --- | --- | --- |
|  | N = 11,652 | N = 7,667 |
| **Demographic Characteristics on Index** |  |  |
| Age, Mean (SD) | 60.8 (12.8) | 60.6 (12.8) |
| Median | 60 | 59 |
| Age category, N (%) |  |  |
| 18-29 | 79 (0.7%) | 67 (0.9%) |
| 30-39 | 411 (3.5%) | 259 (3.4%) |
| 40-49 | 1,548 (13.3%) | 1,019 (13.3%) |
| 50-59 | 3,697 (31.7%) | 2,509 (32.7%) |
| 60-69 | 2,961 (25.4%) | 1,886 (24.6%) |
| 70-79 | 1,880 (16.1%) | 1,252 (16.3%) |
| 80-89 | 949 (8.1%) | 601 (7.8%) |
| 89+ | 127 (1.1%) | 74 (1.0%) |
| Sex, N (%) |  |  |
| Male | 6,468 (55.5%) | 4,174 (54.4%) |
| Female | 5,184 (44.5%) | 3,493 (45.6%) |
| Geographic region, N (%) |  |  |
| Northeast | 1,363 (11.7%) | 962 (12.6%) |
| North Central | 3,543 (30.4%) | 2,338 (30.5%) |
| South | 5,442 (46.7%) | 3,525 (46.0%) |
| West | 1,282 (11.0%) | 826 (10.8%) |
| Unknown | 22 (0.2%) | 16 (0.2%) |
| Insurance plan type, N (%) |  |  |
| Comprehensive/indemnity | 1,893 (16.3%) | 641 (8.4%) |
| EPO/PPO | 5,778 (49.6%) | 4,212 (54.9%) |
| POS/POS with capitation | 721 (6.2%) | 284 (3.7%) |
| HMO | 1,522 (13.1%) | 1,404 (18.3%) |
| CDHP/HDHP | 1,529 (13.1%) | 1,040 (13.6%) |
| Other/Unknown | 209 (1.8%) | 86 (1.1%) |
| Payer type, N (%) |  |  |
| Commercial | 7,741 (66.4%) | 5,211 (68.0%) |
| Medicare | 3,911 (33.6%) | 2,456 (32.0%) |
| **Clinical Characteristics during 12 Month Baseline** |  |  |
| Deyo-Charlson Comorbidity Index, Mean (SD) | 2.8 (1.6) | 2.7 (1.6) |
| Median | 2 | 2 |
| Deyo-Charlson Comorbidity Index, N (%) |  |  |
| 0 | 397 (3.4%) | 290 (3.8%) |
| 1 | 1,689 (14.5%) | 1,303 (17.0%) |
| 2 | 4,456 (38.2%) | 2,845 (37.1%) |
| 3 | 1,759 (15.1%) | 1,093 (14.3%) |
| 4+ | 3,351 (28.8%) | 2,136 (27.9%) |
| Diabetes Complications Severity Index, Mean (SD) | 2.6 (1.8) | 2.5 (1.7) |
| Median | 2 | 2 |
| Type of diabetes diagnosis, N (%) |  |  |
| Diabetes mellitus due to underlying condition | 570 (4.9%) | 236 (3.10%) |
| Drug or chemical induced diabetes mellitus | 11 (0.1%) | 9 (0.10%) |
| Type 1 diabetes mellitus | 1,752 (15.0%) | 773 (10.10%) |
| Type 2 diabetes mellitus | 11,003 (94.4%) | 7,221 (94.20%) |
| Other specified diabetes mellitus | 769 (6.6%) | 307 (4.00%) |
| pDPN-Related Comorbid Conditions, N (%) |  |  |
| Anxiety | 1,021 (8.8%) | 817 (10.7%) |
| Depression | 682 (5.9%) | 434 (5.7%) |
| Cardiovascular disease | 3,442 (29.5%) | 2,157 (28.1%) |
| Cognitive dysfunction | 340 (2.9%) | 190 (2.5%) |
| Vitreous detachment | 459 (3.9%) | 305 (4.0%) |
| Diabetes-related infections^2^ | 2,426 (20.8%) | 1,349 (17.6%) |
| Hypertension | 8,832 (75.8%) | 5,707 (74.4%) |
| Hypoglycaemia | 214 (1.8%) | 110 (1.4%) |
| Lipid disorders (i.e., hyperlipidemia) | 7,830 (67.2%) | 5,260 (68.6%) |
| Nephropathy | 3,148 (27.0%) | 2,046 (26.7%) |
| Obesity | 3,962 (34.0%) | 2,929 (38.2%) |
| Sleep disorders | 2,566 (22.0%) | 1,730 (22.6%) |
| **Treatment Utilization** |  |  |
| Treatment utilization at index, N (%) |  |  |
| Gabapentinoids | 7,270 (62.39%) | 5,226 (68.16%) |
| *Gabapentin* | *6,725 (57.72%)* | *4,767 (62.18%)* |
| *Pregabalin* | *545 (4.68%)* | *459 (5.99%)* |
| SNRI antidepressants | 655 (5.62%) | 527 (6.87%) |
| Tricyclic antidepressants | 422 (3.62%) | 278 (3.63%) |
| Opioids | 2,825 (24.24%) | 1,454 (18.96%) |
| Sodium channel antagonists | 76 (0.65%) | 37 (0.48%) |
| Topical agents | 576 (4.94%) | 269 (3.51%) |
| **Discontinuation of Index Medication within 12 Months Follow-Up** |  |  |
| Patients with discontinuation, N (%) | 9,008 (77.3%) | 5,867 (76.5%) |
| Discontinue during months 0-3, N (%) | 6,691 (57.4%) | 4,212 (54.9%) |
| Discontinue during months 4-6, N (%) | 1,214 (10.4%) | 843 (11.0%) |
| Discontinue during months 7-12, N (%) | 1,103 (9.5%) | 812 (10.6%) |
| Days to discontinuation, Mean (SD) | 73.6 (83.2) | 79.9 (85.7) |
| Median | 30.0 | 30.0 |

^1^ Pre-COVID; patients with follow-up end prior to March 1, 2020, COVID; patients with an index date or after March 1,2020.
^2^ Diabetes-related infections include respiratory infections, urinary tract infections, gastrointestinal and liver infections, soft tissue and bone infections, head and neck infections.
*Abbreviations*: CDHP: Consumer-driven health plan; DPN: diabetic peripheral neuropathy; EPO: Exclusive provider organization; HDHP: High deductible health plan; HMO: Health maintenance organization; N: number of participants; pDPN: painful diabetic peripheral neuropathy; POS: Point of service; PPO: Preferred provider organization; SD: standard deviation; SNRI: Serotonin and norepinephrine reuptake inhibitors.

**References**

1.	Li, T.H., et al., *Impact of the COVID-19 pandemic on treatment for mental health needs: a perspective on service use patterns and expenditures from commercial medical claims data.* BMC Health Serv Res, 2023. **23**(1): p. 163.
